# Advertisers and Advertising

**Published:** 1905-05

**Authors:** 


A Monthly Review of Medicine and Surgery.

*EDITOR:*

WILLIAM WARREN POTTER, M. D.

All communications, whether of a literary or business nature, books for review and exchanges, should be addressed to the editor 284 Franklin St., Buffalo, N. Y.

Vol. XLIV.—LX. MAY, 1905. No. 10

### Advertisers and Advertising.

TO ADVERTISE or not to advertise is a burning question with many dealers, manufacturers, or importers of materials, drugs or preparations in which doctors are interested. Long established houses with large experience usually have adopted a principle regarding the question of advertising from which they are not likely to deviate, unless conditions change in a marked degree.

The business houses which have taken the field more recently are sometimes greatly perplexed as to what is best to be done,—whether to advertise on a large or a limited scale; whether to advertise at all; whether to place their advertising in the hands of an agent or to deal directly with the magazines. These questions are entirely apart from the ethical viewpoint and with which the present discussion has nothing whatever to do. We are just now concerned with the consideration of the best method for the advertiser, and in dealing with it we disclaim absolutely any personal or selfish aim, but present a few thoughts that have arisen from an observation extending over a period of several years.

We recognise that conditions are changeable and ever changing, that what will meet the necessities of today may be unsuited for the demands of tomorrow, and that no rule can be made so hard and fast as to apply to everybody. The discussion of some of these problems formed an interesting feature of a recent dinner of the Advertisers' Club of Western New York, at the Hotel Lafayette, Buffalo, and perhaps we cannot present the argument in any better fashion than to quote from the remarks of Mr. James

Rodgers, of Harper Brothers, who was one of the principal guests of the club. Mr. Rodgers has been at the advertising end of the Harpers's publications for 31 years and was full of enthusiasm about them. He exhibited and compared copies of the magazine every ten years from the first in 1850 to the May issue for 1905. He told much history, read some of the old advertisements, quoted the monthly's first editorial announcement of the magazine's intention.

"As to the comparative values of different size advertisements," said he, "I say that the half-page ad is three times as valuable as the quarter page, the full page four times as effective as the half, and two pages facing each other are five times as effective as one page."

He strongly commended "the right kind of an illustration." Various trademarks, features and pictures, now famous through persistent advertising, were mentioned and criticised. Some conspicuous successes were mentioned, with their advertising campaign outlined, also some business failures or comparative failures, with their advertising campaign.

The case of a great baking powder company then was brought up and Mr. Rodgers said that after years of thorough and continued advertising, the president called in the publicity chief and said: "Here, we're paying $650,000 a year advertising. It's too much. Cut it down as much as possible."

The publicist said: "No, don't do that. I know the end will be disastrous. Do not dare to take such a step. If you feel like experimenting begin with a part of your business, not the whole. Cut out, if you want to, the advertising in two Western States for a year and see what happens. If you're right about momentum carrying you along from now on, you can cut down the rest of the advertising.

The experiment was tried. There was a tremendous falling off of the baking-powder company's business in the two states and it was only after years of most extensive advertising in the same two states that a business was again built up there anywhere near its former magnitude.

Continuing, Harper's, said he, had only 116 pages of advertising in the whole twelve issues of 1884. Twenty-one years later, there are 153 pages of ads in one month's issue. From those and other comparisons he made, he concluded that railroading and other forms of profitable activity have not progressed in the same great degree that had the development of the magazine. Answering a question, Mr. Rodgers said he would not give a snap for circulation as compared with knowledge of the kind of people the medium reaches.

